# Baculovirus-mediated promoter assay and transcriptional analysis of white spot syndrome virus *orf427 *gene

**DOI:** 10.1186/1743-422X-2-71

**Published:** 2005-08-23

**Authors:** Liqun Lu, Hai Wang, Ivanus Manopo, Li Yu, Jimmy Kwang

**Affiliations:** 1 Animal health biotechnology unit, Temasek life sciences laboratory, 1 Research Link, National University of Singapore, 117604, Singapore

## Abstract

**Background:**

White spot syndrome virus (WSSV) is an important pathogen of the penaeid shrimp with high mortalities. In previous reports, *Orf427 *of WSSV is characterized as one of the three major latency-associated genes of WSSV. Here, we were interested to analyze the promoter of *orf427 *and its expression during viral pathogenesis.

**Results:**

*in situ *hybridization revealed that *orf427 *was transcribed in all the infected tissues during viral lytic infection and the translational product can be detected from the infected shrimp. A time-course RT-PCR analysis indicated that transcriptional products of *orf427 *could only be detected after 6 h post virus inoculation. Furthermore, a baculovirus-mediated promoter analysis indicated that the promoter of *orf427 *failed to express the EGFP reporter gene in both insect SF9 cells and primary shrimp cells.

**Conclusion:**

Our data suggested that latency-related *orf427 *might not play an important role in activating virus replication from latent phase due to its late transcription during the lytic infection.

## Background

White spot syndrome virus (WSSV) was assigned to the genus *Whispovirus *belonging to new family *Nimaviridae *in the universal database of ICTV (International Committee of Taxonomy of Viruses, ). WSSV is probably the most important pathogen of the cultured penaeid shrimp resulting in high mortalities [1]. Even though WSSV represents one of the largest known animal viruses with a 305 kb double-stranded circular DNA genome, most of the putative 185 ORFs bear no homology to known genes in the GenBank [2,3]. The technical difficulty in characterization of the WSSV ORFs lies mainly in the lack of established shrimp cell lines for *in vitro *reproduction of the virus [4]. During viral lytic infection, just as other DNA viruses, the genes encoded by WSSV can be classified as immediately early, delayed early, late and very late genes. Most, if not all, immediate-early genes encode transcriptional regulation proteins. They are distinguished from other viral genes by the fact that their transcription is independent of prior viral gene product expressed in transient assays [5]. Although during the last decade, intensive efforts have been undertaken for characterization of the structural protein genes and a few non-structural protein genes that show homology to known sequences in the databases, little is known about the molecular mechanisms underlying the WSSV life cycle and mode of infection.

Recently, three viral transcripts (*Orf427*, *Orf151 *and *Orf366*) and their corresponding DNA sequence have been detected in both specific-pathogen-free (SPF) shrimps and WSSV-infected shrimps through a WSSV-specific DNA microarray study. From this study, *Orf427*, *Orf151 *and *Orf366 *were determined to be latency-associated genes of WSSV [6]. These data suggest that WSSV remains latent in healthy shrimps. In a similar global analysis, three immediately early (IE) genes (*ie1*, *ie2*, and *ie3*) of WSSV were identified in infected shrimps [7]. Identification of the IE genes and latency-associated genes can lead to better understanding of the life cycle of WSSV, shedding light on the molecular mechanisms in WSSV-induced mortality. In a previous study, we have found that latency-related ORF427 interacted with a shrimp protein phosphatase (PPs) [8]. To further characterize the *orf427 *gene, we were interested to analyze the promoter of *orf427 *and its expression during viral pathogenesis.

## Results

**Figure 1:**
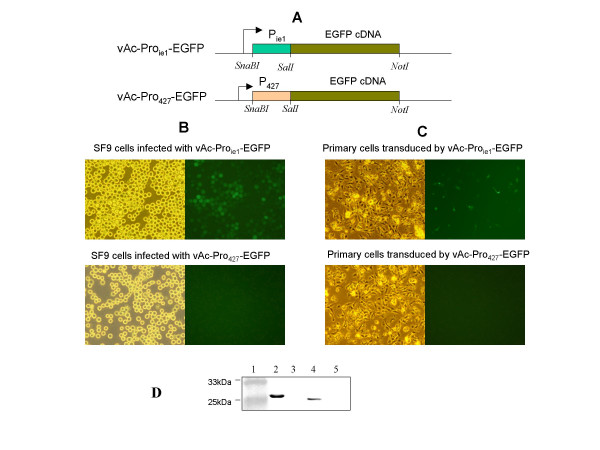
Baculovirus-mediated promoter analysis of *orf427 *compared with immediate-early gene *ie1*. **A) **Genomic organization of vAc-Proie1-EGFP and vAc-Pro427-EGFP. Pie1, promoter of *ie1 *gene; P427, promoter of *orf427*. Recombinant baculoviruses were constructed using the Bac-To-Bac system (Invitrogen). The EGFP-expressing cassettes were first cloned into the pFastBac1 shuttle vector at the indicated restriction sites and then integrated into the bacmid genome through site-specific transposition. **B) **Promoter activity of *orf427 *and *ie1 *gene in insect SF9 cells. Brightfield and EGFP fluorescence signals in SF9 cells infected with vAc-Proie1-EGFP and vAc-Pro427-EGFP at m.o.i of 10, respectively. **C) **Promoter activity of *orf427 *and *ie1 *gene in primary shrimp cells. Brightfield and EGFP fluorescence signals in primary shrimp cells transduced with vAc-Proie1-EGFP and vAc-Pro427-EGFP at m.o.i of 100, respectively. **D) **Western blot assay to confirm the expression of GFP in virus-infected or transducted cells. 1. Protein marker; 2. vAc-Proie1-EGFP infected SF9 cells; 3. vAc-Pro427-EGFP infected SF9 cells; 4. vAc-Proie1-EGFP transduced shrimp primary cells; 5. vAc-Pro427-EGFP transduced primary shrimp cells.

To investigate whether promoter of *orf427 *is active without the existence of other viral proteins in the host cells, we tried to establish *in vitro *culture of fragments from lymphoid organ as reported previously [9]. However, the primary shrimp cells were very sensitive to standard liposome-based transfection reagents. Thus, for the promoter analysis, we employed a transduction method mediated by baculovirus [10]. Recombinant baculoviruses bearing EGFP-expressing cassettes were produced according to pFASTBac1 manufacturer instructions (Invitrogen) (Fig. 1A). Budded virus from insect cell culture medium was concentrated by ultrafiltration and infectious titers of both stock viruses were determined by plaque assay and adjusted to be 10^10 ^plaque-forming units (PFU)/ml.

Infection of SF9 cells and transduction of shrimp primary cells with the recombinant baculovirses were carried out at a MOI of 10 and 100, respectively. As expected, the *ie1 *promoter drove the expression of the *egfp *reporter gene in both insect SF9 and the primary shrimp cells, as demonstrated by direct light and fluorescence microscopy; while the *orf427 *promoter didn't express *egfp *to a detectable level in either cell type (Fig. 1B and 1C). The expression of GFP could be confirmed in both cells through immunoblot assay using monoclonal anti-GFP antibody (Fig. 1D). We also noticed that the primary shrimp cells could only be transduced at a low percentage of about 5% (Fig. 1C).

**Figure 2:**
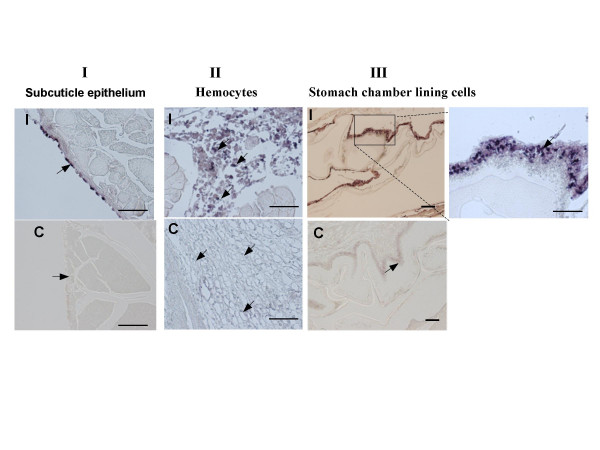
Detection of *orf427 *mRNA in different tissue sections from WSSV-infected shrimp by *in situ *hybridization with specific *orf427 *antisense riboprobe. I: WSSV-infected shrimp; C: non-infected shrimp; the short bar is about 30 μm in length. **1) **Section of subcuticle epithelium; **2) **Section of hemocytes; **3) **Section of stomach chamber lining cells.

**Figure 3:**
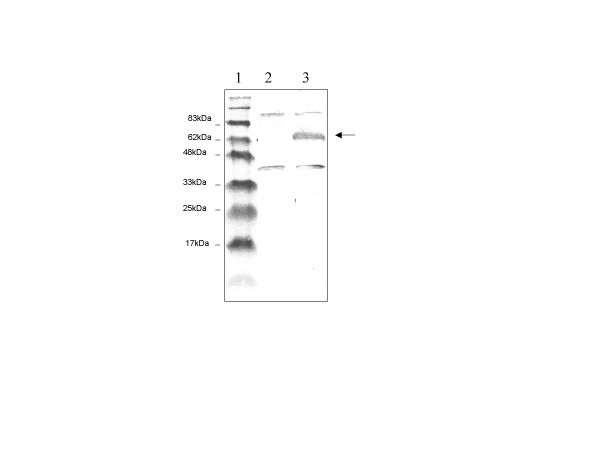
**Detection of ORF427 in infected shrimp through Western blot analysis. **Western blotting analysis for detection of the endogenic ORF427 in infected shrimp cells. Polyclonal antibody toward Orf427 was raised using the bacterially expressed partial Orf427 as antigen from Guinea pigs. 1. Protein marker; 2, total shrimp cellular extracts sampled from normal shrimp; 3, total shrimp cellular extracts sampled from WSSV-infected shrimp.

In most cases, viruses establish latency in specific tissue(s). To test whether *orf427 *is transcribed only in specific latency sites or in all the tissues that support viral infection, *in situ *hybridization was performed on paraffin embedded tissue sections from shrimps at late infection (4 days after viral inoculation) using DIG-labeled antisense RNA probes specific for *orf427*. Results shown in fig. 2 indicated that in contrast to the control shrimp sections, *orf427 *was extensively transcribed in all the WSSV infected tissue sections including subcuticular epithelium cells (Fig. 2I), hemocytes lodged in the connective tissues (Fig. 2II), and stomach chamber lining cells (Fig. 2III). Also, we expressed and purified partial fragment of ORF427 in a GST-fusion form. Protein purity of the purified protein was more than 90% as judged by SDS-PAGE (figure not shown). Polyclonal antibody was developed by injection of the protein into Guinea pigs. ORF427 can be detected from homogenized infected shrimps through immunoblot assay using the anti-ORF427 antibody (Fig. 3).

**Figure 4:**
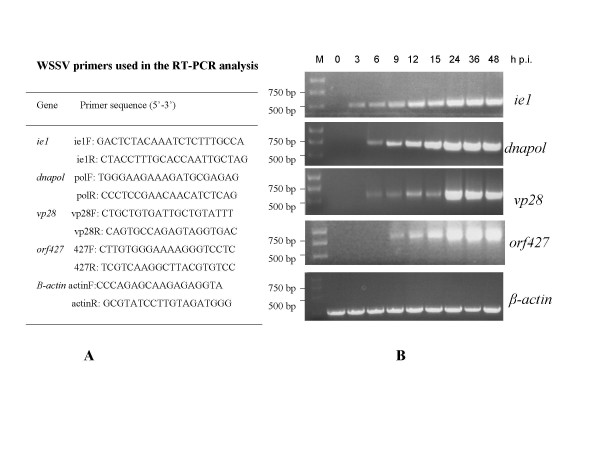
Time course RT-PCR analysis of *orf427 *during viral pathogenesis. **A) **Gene specific primer sets used in the RT-PCR analysis as previously reported [6,7]. **B) **Agarose gel electrophoresis of RT-PCR products. Total RNA was sampled at the indicated time points post infection and RT-PCR was performed using primer sets specific for *ie1*, *dnapol*, *vp28*, *orf427*, and β-actin gene, individually. M: kb DNA ladder from Stratagene.

In order to determine whether *orf427 *is transcribed in the early phase during viral lytic infection, we employed a RT-PCR approach to detect the transcriptional products of *orf427*. The sequences of the primers used are shown in Fig. 4A. *P. monodon *shrimps challenged through intramuscular injection with WSSV were sampled at different time points after viral inoculation, and total RNAs were extracted from the shrimp heads for RT-PCR analysis. As controls, fragments corresponding to the WSSV immediately early gene *ie1 *[7], delayed early gene *dnapol *[11], and late gene *vp28 *[12], were also amplified from the same RNA samples. A shrimp β-actin primer set was used as an internal control for RNA quality and amplification efficiency. Our results show that *orf427 *is only transcribed after 6 h post infection (Fig. 4B), which is at the late phase during viral lytic infection. As expected, *ie1 *can be detected from 3 h p.i., while *dnapol *and *vp28 *can be detected from 6 h p.i. (Fig. 4B).

## Discussion

Establishment and maintenance of latency in the host after primary infection have been investigated in some well-studied DNA viruses such as: herpes simplex virus (HSV) [13], human herpesvirus (HHV) [14], cytomegalovirus (CMV) [15], and Epstein-Barr virus [16]. However, the molecular mechanisms that control virus latency and reactivation remain to be elucidated. Because of problems associated with conducting molecular studies in animals, it has proven difficult for investigators to move beyond phenomenal description and identification of latency-associated transcripts (LATs). Most of the characterized LATs were expressed at low levels during lytic replication but were major transcripts during latent infection, and their functions were not understood. These include a set of latency-associated transcripts from the HHV-6 IE-A region [17], a set of genes controlled by the Qp promoter of Epstein-Barr virus [16], and latency-associated transcripts from both DNA strands in the ie1/ie2 region of CMV [15]. U94 gene of HHV-6 is one of the better-characterized LATs. U94 protein acts as a transactivator by binding to a transcription factor and enables the establishment and/or maintenance of latent infection at the primary infection site like monocytes and early bone marrow progenitor cells [18]. Our data indicate that *orf427 *is a very late gene during viral lytic infection, and this correlates with the finding that ORF427 is not a transcriptional regulator, but a protein phosphatase-interacting protein [8].

Most recently, nuclear protein phosphatase-1 was reported to regulate HIV-1 transcription both *in vitro *and *in vivo *[19]. Primary functional dissection of *Orf427 *suggests that *orf427 *most likely encodes a calcium-binding regulator of shrimp protein phosphatase, with the C terminus responsible for the binding of PPs (data not shown). This suggests that *orf427 *is not necessary for viral reactivation and only contributes to maintaining viral latency by affecting the function of shrimp protein phosphatase. Similarly, the LAT gene of HSV-1 has been shown to be dispensable for viral reactivation from latently infected mouse dorsal root ganglia cultured *in vitro *[20].

The development of a continuous shrimp cell line *in vitro *is urgently required for further characterization of WSSV infection at the molecular and cellular levels. In recent years, encouraging progress has been made in shrimp cell culture using conventional primary culture techniques. Several investigators have reported that WSSV infects the primary cultures of lymphoid organs from the black tiger shrimp, *P. monodon*; however, recent findings suggest that the replication of WSSV in lymphoid organ primary cell is low [4,9,21]. Besides this, the primary cell couldn't be transfected with common liposome methods. We thus took alternative approach to monitor the gene expression in the primary shrimp cells. Recently AcMNPV (*Autographa californica *multiple nucleopolyhedrovirus), containing an appropriate eukaryotic promoter, was used to efficiently transfer and express foreign genes in a variety of mammalian cells and several animal models [22]. Considering that shrimp is more phylogenically related to arthropods, the natural host of AcMNPV, we employed recombinant baculovirus-mediated transduction to introduce foreign genes into the primary shrimp cells. As expected, the primary shrimp cells were transduced in our experiments; and the low transduction efficiency might be due to the possible inhibition effect of L15 medium on the attachment of baculovirus to the cell membrane (for example, the pH value of medium for insect cells to amplify baculovirus is 6.8, while the pH value of L15 medium is above 7.0). The transduction efficiency might be significantly increased by using VSV-G-containing baculovirus as gene delivery vehicle [10]. The successful transduction of cultured shrimp cells with recombinant baculovirus may pave the way for the development of baculovirus-based vaccines for the shrimp farming industry.

## Conclusion

The data presented here demonstrates that latency-associated *Orf427 *is only transcribed in the very late phase during viral lytic infection. In contrast to immediately early promoters, the promoter of *orf427 *couldn't drive the expression of an *egfp *reporter gene independently. Our data suggest that as a very late protein during viral lytic infection, ORF427 might only function in maintaining WSSV in the latent phase but is not required for virus reactivation.

## Materials and methods

### Virus, shrimp, and cells

WSSV used in this study was isolated from *Penaeus monodon *shrimps, which were imported from Indonesia. Purification of the virus was performed as previously described [6]. *P. monodon *shrimps challenged through intramuscular injection were sampled at different time points postinfection and immediately frozen and stored at -80°C. Adult *P. monodon *shrimps weighing approximately 30–100 g were used for primary cell culture. Monolayer cell cultures from minced fragments of lymphoid tissue were established as described by Chen [9]. Primary cells were maintained in 2 × L15 medium from Invitrogen. Insect SF9 cells (Invitrogen) were maintained and propagated in SF-900II serum-free medium (Invitrogen). Infection of SF9 cells and transduction of foreign genes into shrimp primary cells were performed as previously described [10].

### Construction of recombinant baculoviruses

The *ie1 *basic promoter region from -1 to -512 was amplified using primer set of 5'-TCCCTACGTATCAATTTTATGTGGCTAATGGAGA-3' and 5'-ACGCGTCGA CCTTGAGTGGAGAGAGAGCTAGTTATAA-3' [7]. To make sure that the selected promoter region contained the full *orf427 *promoter, the upstream sequence of *orf427*, starting from -1 to -3500, was PCR-amplified from WSSV genome with primer set of 5'-TCCCTACGTATGGGTCAGAAAAGAACCC-3' and 5'-ACGCGTCGACATC TCAAAGGTTGCCAATGACCAACAT-3'. Both promoters were digested with *SnaBI *and *SalI*, and inserted into the corresponding sites of shuttle vector pFastBac1 (Invitrogen). The EGFP cDNA was first cut with *SalI *and *NotI *from the pEGFP-N1 vector (Clontech), followed by insertion into the pFASTBac1 vector bearing the promoter sequence of *orf427 *or *ie1 *gene. Recombinant baculoviruses bearing the EGFP-expression cassette were constructed according to the Bac-To-Bac protocol (Invitrogen). The infectious titers of the recombinant baculoviruses were determined by plaque assay with SF9 cells.

### Development of polyclonal antibody and Western blot analysis

The C terminal partial fragment amplified from *orf427 *template using primer pair of 5'-CGGGATCCGTTAGAGCTTCAAAGGTGGA-3' and 5'-ACGCGTCGAC TTATTTTCCTTGATCTAGAG-3' was inserted into the pGEX4T-3 vector at BamH1 and Sal I site. The partial ORF427 was expressed and purified in *E. coli *as a glutathione S-transfererase (GST) fusion protein according to manufacturer's protol (Amersham Pharmacia). SPF Guinea pigs were immunized and specific antisera were prepared using standard procedures. Homogenized protein mixtures from infected shrimp or virus-infected cells were harvested and subjected to sodium dodecyl sulfate (SDS)-polyacrylamide gel electrophoresis (PAGE). Immunoblot analysis was performed according to standard protocol [23].

### In situ hybridization

*In situ *hybridization was performed on paraffin embedded tissue sections using a DIG-labeled antisense RNA probes. Both WSSV-free shrimps and WSSV-infected shrimps were fixed in 4% (W/V) paraformaldehyde (PFA)-PBS, dehydrated, and embedded in paraffin. Sections of 6 μm in thickness were made and attached to 3-aminopropyltriethoxy-silane-coated slides. DIG-labeled antisense riboprobe specific for *orf427 *was synthesized by *in vitro *transcription using T7 RNA polymerase (Stratagene) and 10 × Dig labeling mix (Roche). The transcription template was PCR amplified from *orf427 *with a primer set of 5'-TAATACGACTCACTATAGGGCGCACCAGAAGAAAGGGTCT-3', and 5'-AAGGAAAC CATCGAGGCCTT-3'. The T7 promoter sequence was flanked at the 5' of the reverse primer. Hybridization was performed in 50% formamide and 5 × SSC in a humified chamber at 60°C for 14–16 h (the background is too high at 50°C in our hybridization system). The hybridization was visualized by using alkaline phosphatase-conjugated anti-digoxigenin antibody.

### RT-PCR analysis

Total RNA was extracted from heads of the WSSV-infected shrimps using TRIzol-LS reagent (Life Technologies). For RT-PCR, an aliquot of total RNA (20 μg) was treated with 200 U of RNase-free DNase I (Gibco BRL) at 37°C for 30 min to remove residual DNA. First strand cDNA synthesis was performed using the oligo-dT primer, and 2 μl of the cDNA was subjected to PCR in a 50 μl reaction mixture.

## Competing interests

The author(s) declare that they have no competing interests.

## Authors' contributions

Jimmy Kwang designed the study and critically reviewed the manuscript. Liqun Lu performed all the experiments and wrote the manuscript. Wang Hai helped perform *in situ *hybridization. Ivanus Manopo helped prepare shrimp primary cells and critically review the manuscript. Yu Li constructed and tested the plasmid containing WSSV ie1 promoter.
